# Integrating disease mapping and flexible scan statistics to identify and visualize spatial clusters

**DOI:** 10.1371/journal.pone.0353971

**Published:** 2026-08-05

**Authors:** Lina Wang, Haoqi Hu, Yaru Li, Danfei Zhang, Xiang Li, Zhengbin Zhang

**Affiliations:** 1 School of Computer Science and Artificial Intelligence, Zhengzhou University of Light Industry, Zhengzhou, Henan, China; 2 Institute of Surveying and Mapping, Information Engineering University, Zhengzhou, Henan, China; 3 Tuberculosis Prevention and Control Office, Wuhan Pulmonary Hospital (Wuhan Institute for Tuberculosis Control), Wuhan, Hubei, China; SIT: Singapore Institute of Technology, SINGAPORE

## Abstract

Accurately characterizing the spatial distribution of diseases is essential for understanding etiology and guiding targeted public health interventions. This study develops an analytical framework integrating disease mapping and spatial scan statistics to identify and validate the spatial clustering patterns of diseases. The framework follows an “exploration–validation–refinement” workflow, combining qualitative visualization with quantitative spatial analysis. Choropleth maps intuitively depict the overall distribution of diseases, providing a basis for identifying potential high-risk areas; the flexible scan statistics (FleXScan) method detects statistically significant clusters; and dot cartograms further reveal internal heterogeneity within the most likely clusters by controlling for population density. This study validated the framework using both synthetic data and real measles case data. The results indicate that the framework substantially improves the accuracy and reliability of cluster detection, facilitates the localization of local high-risk cores, and provides actionable insights for precision disease control.

## 1. Introduction

The occurrence and spread of diseases are closely associated with population characteristics, natural environments, and social conditions, which often exhibit distinct spatial patterns in their distribution [1]. Accurately characterizing the spatial distribution of diseases and revealing spatiotemporal variations in risk are essential for understanding disease mechanisms and transmission pathways, and for informing public health interventions and resource allocation [2].

Disease mapping serves as a crucial tool for revealing spatial patterns of diseases [3–4]. As early as the 19th century, John Snow’s cholera map of London illustrated a direct link between the outbreak area and the contaminated Broad Street water pump, and the removal of the pump handle effectively curtailed the epidemic. This landmark case is widely recognized as the origin of modern disease mapping, marking the transition from merely recording disease occurrences to actively exploring the relationships between geographic environments and disease etiology [3,5,6]. Since the 20th century, advances in statistical methods and geographic information systems (GIS) have facilitated quantitative and dynamic disease mapping, enabling data integration and visualization that support disease surveillance and control [7].

However, traditional disease maps largely rely on visual interpretation. While they can intuitively illustrate spatial differences, they cannot determine whether these differences are statistically significant or merely the result of random variation. In particular, choropleth maps may overemphasize the influence of geographic area, potentially leading to visual bias, which may mislead interpretation: regions with large geographic areas but low incidence may appear overly prominent, whereas small regions with high incidence may be visually understated. Such discrepancies between statistical values and geographic representation can mislead interpretation [3,8–15]. In contrast, spatial clustering analysis uses statistical testing to identify significantly high-risk clusters, providing quantitative verification for visually observed spatial patterns and allowing more accurate differentiation between true clusters and random variation.

Spatial clustering analysis aims to determine whether a disease exhibits spatial aggregation and to locate the corresponding cluster areas. The methods can be broadly categorized into global and local approaches. Global methods, such as Global Moran’s I [16], are used to evaluate the overall spatial autocorrelation of disease distribution but cannot precisely identify cluster locations or extents of clusters. In contrast, local methods can detect high-risk clusters and assess their statistical significance. Among these, the spatial scan statistic proposed by Kulldorff [17–18] has been the most widely applied and has been extensively used to analyze the spatial and spatiotemporal distribution characteristics of various diseases, including cancer [19], mumps [20], tuberculosis [21–22], and COVID-19 [23]. The major advantage of this method lies in its ability to automatically identify statistically significant high-risk clusters, delineate their spatial extent, and estimate relative risk values. However, it is limited to circular or elliptical scanning windows and thus cannot accurately detect clusters with irregular shapes. To overcome this limitation, Tango et al. proposed the flexibly shaped spatial scan statistic (FleXScan), which constructs irregular scanning windows through a neighborhood expansion strategy, thereby enabling identified clusters to better conform to actual geographic boundaries [24–26].

However, results may vary across different methods, and even within the same method, outcomes are highly sensitive to parameter settings. Such sensitivity can affect the stability and accuracy of cluster detection when using real disease data. Furthermore, most previous studies have focused primarily on cluster detection itself, with limited systematic comparison or joint validation with visualization outputs, which restricts the interpretability and reliability of the findings.

Therefore, integrating disease visualization with spatial clustering analysis is of considerable importance. Visualization can intuitively reveal spatial patterns of disease, providing preliminary insights into potential clusters, while clustering analysis enables statistical validation and quantitative assessment. The combination of these approaches forms an “exploration–validation–refinement” analytical framework, which not only supports assessment of cluster authenticity but also enhances the scientific rigor and interpretability of the results.

To evaluate the effectiveness of this framework, this study draws on both synthetic data and real measles data from Henan. Choropleth maps, dot cartograms and the FleXScan method are applied to investigate and validate spatial disease patterns from a perspective that integrates visualization and clustering analysis. Dot cartograms are contiguous area cartograms that display spatial point data as a dot-density pattern [27]. By comparing the outcomes of these methods, the study aims to reveal both consistencies and discrepancies in disease spatial patterns, providing a reference for understanding disease clustering mechanisms and informing potential control strategies.

## 2. Overall framework

**Fig 1:**
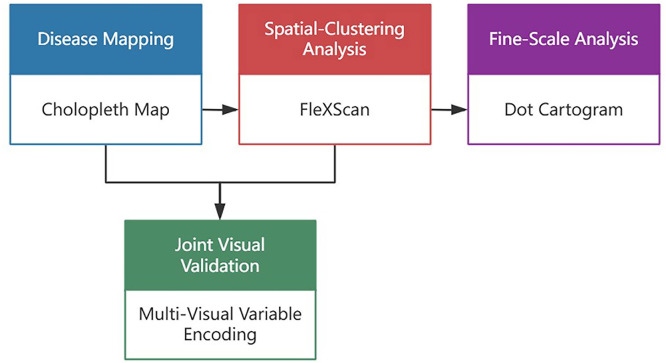
Framework of the study.

This study establishes a comprehensive analytical framework that integrates disease mapping with spatial clustering analysis, forming an “exploration–validation–refinement” workflow (as shown in Fig 1). First, choropleth maps were used to visually depict the spatial distribution of the disease, enabling preliminary identification of potential high-risk regions. Second, spatial clustering analysis was conducted using the FleXScan method to identify statistically significant high-risk clusters. Subsequently, the clustering results were visualized and overlaid on the incidence maps using multiple visual variables, where different visual variables were employed to simultaneously represent incidence rates and the boundaries of high-risk clusters, achieving combined visual validation of spatial patterns. Finally, based on the raw case point data, dot cartograms adjusted for population density were used to analyze the internal spatial heterogeneity within high-risk clusters, providing a spatial basis for targeted, precise disease control interventions.

## 3. Methods

### 3.1 Choropleth map

In disease data visualization, methods such as standardizing incidence rates or population weighting are commonly used to eliminate the effects of population distribution, allowing for a more objective presentation of the spatial patterns of disease risk. Choropleth maps are a widely used method in epidemiology to represent rate data (e.g., incidence or mortality rates) [28]. This method uses variations in color intensity to represent differences in values across regions, providing a visual means of revealing the spatial distribution and regional differences in disease risk.

However, choropleth maps have several limitations. In particular, the choice of classification method and the number of classes can substantially influence the visual representation and interpretability of the results, requiring careful consideration based on the data distribution and research objectives. Choropleth maps are also affected by the selection of spatial units and scale, a phenomenon known as the modifiable areal unit problem (MAUP). Different spatial divisions can lead to discrepancies in observed disease patterns, affecting the stability and reliability of the analysis.

### 3.2 FleXScan method

In spatial epidemiology, accurately identifying disease clusters is crucial for guiding public health interventions and optimizing resource allocation. The FleXScan method is an extension of traditional spatial scan statistics, capable of detecting disease clusters of irregular shapes, thereby overcoming the limitations of circular or elliptical scanning windows and better accommodating complex real-world geographic structures.

The method consists of three main steps:

First, the scanning window is defined through neighborhood expansion. The FleXScan method does not preset the shape of the window; instead, it starts from a given spatial unit and gradually includes adjacent regions through a neighborhood expansion strategy to form an irregular scanning area. The key parameter K represents the pre-set maximum number of sub-regions included in the scanning window. As K increases, the number of scanning windows grows exponentially, increasing computational load, but also enhancing the likelihood of identifying true spatial clusters. In this study, a restricted version of FleXScan method was used, with K set to 30 to improve cluster detection capability.

Second, clustering intensity is assessed using statistical modeling and the log-likelihood ratio (LLR). FleXScan method can employ either a Binomial or Poisson model depending on the characteristics of the data. The hypotheses are defined as follows:

**Null hypothesis:** The incidence rates inside and outside the scanning window are equal.
**Alternative hypothesis**: There exists at least one scanning window in which the incidence rate inside the window is higher than outside.

For each candidate window, the LLR is calculated to measure the difference in incidence between the interior and exterior. A higher LLR indicates a more pronounced difference and stronger evidence of clustering. The window with the maximum LLR is identified as the most likely cluster (MLC).

Finally, the statistical significance of the detected clusters is evaluated using Monte Carlo simulation. Random datasets are generated under the null hypothesis, and LLR values are recalculated for each simulation (e.g., 999 iterations). Observed LLR values are then compared with the simulated distribution to compute a p-value. Clusters with p-values below a predefined threshold (e.g., 0.05) are considered statistically significant, providing robust support for the identified spatial clustering patterns.

### 3.3 Dot cartogram

Dot cartograms combine traditional dot maps with area cartograms to provide a more intuitive representation of the spatial distribution and clustering of disease cases. Traditional dot maps display the absolute number of cases but do not account for population density, and may lead to visual congestion of case locations in high-density areas. Choropleth maps, in contrast, reflect population-adjusted incidence rates but rely on predefined administrative boundaries, potentially masking cross-boundary clustering.

The core principle of a dot cartogram is to distort geographic space so that the area of each region is proportional to its population, thereby creating a “population-equalized space,” while simultaneously reprojecting case points according to the same geometric transformation. In the dot cartogram, point density reflects the true distribution of disease rather than differences in population: densely populated regions are expanded, causing points to appear more dispersed, whereas sparsely populated regions are compressed, concentrating points. This approach more accurately depicts spatial patterns of disease while reducing the direct spatial identifiability of individual case locations [6,27]. In this study, area cartograms were generated with the ArcGIS Cartogram Tool plugin, which implements the transformation using a diffusion-based algorithm [29–30].

Dot cartograms have been increasingly applied in epidemiology and public health research. Early theoretical and methodological studies laid the foundation for their use in spatial cluster analysis [8,31–36]. More recent applications include visualizing the spatial distribution of Campylobacter spp. in raw chicken [28], as well as detecting outbreaks such as Q fever in the Netherlands and pertussis in Germany [27]. Adjusted point densities in these examples facilitate clearer identification of spatial clustering and potential outbreak regions.

It should be noted that dot cartograms are primarily exploratory or heuristic visualization tools, intended to aid researchers in detecting potential spatial clusters and local patterns rather than to provide precise quantitative results. The inherent spatial distortion may obscure precise geographic locations, which can limit spatial interpretability and geographic readability.

## 4. Results

### 4.1 Analysis of synthetic data

#### 4.1.1 Synthetic data.

**Fig 2:**
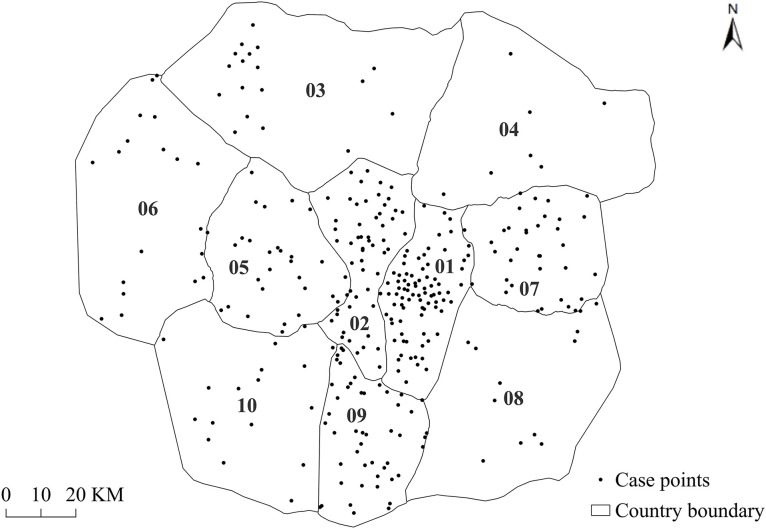
Dot map of disease cases in the synthetic data.

A synthetic dataset consisting of 10 regions was constructed for methodological and visualization demonstration purposes. The regions were used as a synthetic spatial framework. The associated data do not represent actual disease events. Case points within each region were randomly generated using the “Random Points in Polygon” tool in QGIS under a spatially uniform random distribution (as shown in Fig 2), and no clusters were explicitly predefined in the dataset.

To ensure reproducibility, all data used in the study, including the geographic boundary data and their corresponding attribute data, are provided as a Shapefile dataset in the supplementary materials.

#### 4.1.2 Results for synthetic data.

**Fig 3:**
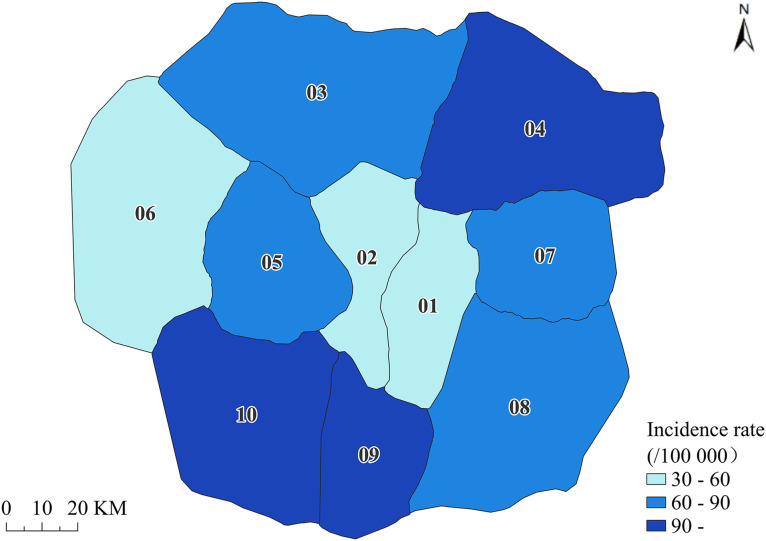
Choropleth map of incidence distribution based on the synthetic data.

A blue color scheme was adopted to facilitate joint visualization with the spatial clustering results and to enhance regional contrast. The incidence values were first classified into three levels based on the natural breaks method, with minor adjustments according to the data distribution. Subsequently, color intensity was used to represent incidence levels, where higher incidence areas are depicted with darker shades of blue and lower incidence areas with lighter shades, thereby improving the visual differentiation of spatial patterns. The resulting incidence choropleth map for the synthetic data is presented in Fig 3.

The FleXScan method was applied to the synthetic data to perform spatial clustering analysis, identifying one statistically significant high-incidence cluster (p < 0.05). Table 1 presents the detected cluster.

**Table 1:** Results of spatial scan statistics for the synthetic data.

Cluster level	Number of sub-regions	Number of cases	Expected cases	Risk population	Log-likelihood ratio	Relative risk	p-value
1	2	65	30.41	54955	16.91	2.14	0.001

**Fig 4:**
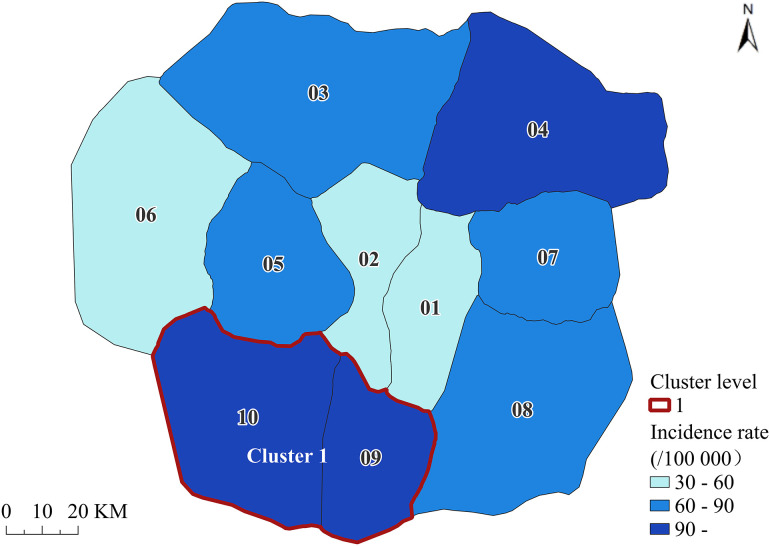
FleXScan-detected cluster on the choropleth map for joint validation.

The disease cluster results were overlaid onto the incidence choropleth map, with varying intensities of red boundary lines representing clustering significance, providing a clear contrast with the incidence distribution (Fig 4). Overall, the high-incidence clusters detected by FleXScan largely corresponded to the high-incidence areas on the map. However, Region 04, despite exhibiting a relatively high incidence rate, was not identified as a statistically significant cluster. This suggests that the observed high incidence in this area may be attributable to random fluctuations or sporadic distribution, as the number of cases and the population base are both relatively small, preventing it from meeting the significance threshold.

**Fig 5:**
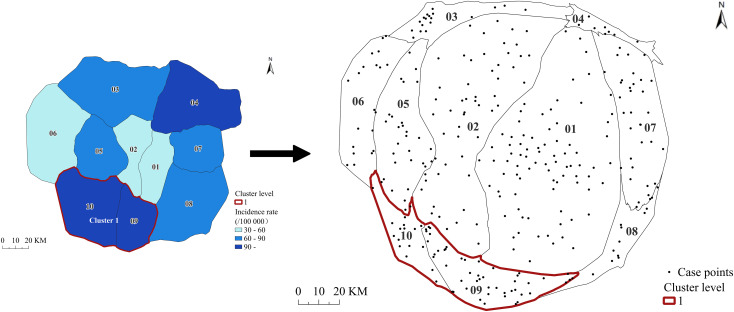
Dot cartogram of simulated disease cases.

Fig 5 presents the dot cartogram corresponding to the synthetic data, in which the area of each region is adjusted according to its population to homogenize population density, allowing the distribution of case points to more accurately reflect the spatial pattern of the disease. In the traditional dot map (as shown in Fig 2), the case points in Regions 01 and 02 appear densely clustered, primarily due to population distribution. After population homogenization in the dot cartogram, the case points in these regions are more evenly distributed. Meanwhile, Region 04, with a relatively low population base, is represented by a smaller area, yet the case points appear more concentrated. These observations indicate that the dot cartogram effectively removes the influence of population density, providing a visually more accurate representation of disease spatial distribution.

Based on the spatial clustering analysis described above, a more detailed examination was conducted for the MLC. Cluster 1 includes regions 09 and 10, which have relatively low population density. As a result, the overall area of the cluster is reduced, and the case points appear more densely distributed visually. These observations highlight the internal heterogeneity within the cluster and provide a basis for identifying local high-risk cores and implementing targeted interventions.

### 4.2 Analysis of real measles data

#### 4.2.1 Real measles data.

The present study utilized county-level measles case data in Henan Province, China, for May 2009, obtained from the S2 Appendix of Reference [37]. Henan Province comprises 157 county-level administrative units. During this period, a total of 1,371 measles cases were reported among a population of 91,669,661, corresponding to an annual incidence rate of 17.6 per 100,000 population. The original data provided the number of cases in each county and the township of each case but lacked precise spatial coordinates for individual cases. Given the requirement for spatial analysis of disease points in this study, cases were randomly distributed within each township according to the number of cases to construct point-level disease data. Considering the analysis was conducted at the provincial scale, this approach has a negligible impact on the visual representation of the spatial distribution. The generated case points do not contain any personally identifiable information.

#### 4.2.2 Results for real measles data.

**Fig 6:**
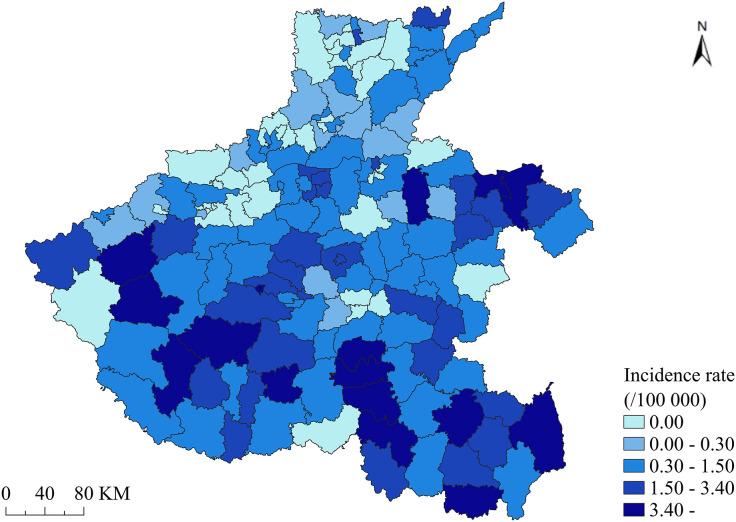
Choropleth map of incidence distribution based on the real measles dada.

The choropleth map of incidence distribution for the real measles case data in Henan Province is presented in Fig 6. Incidence values were classified into five levels, clearly illustrating the spatial distribution patterns across regions.

**Fig 7:**
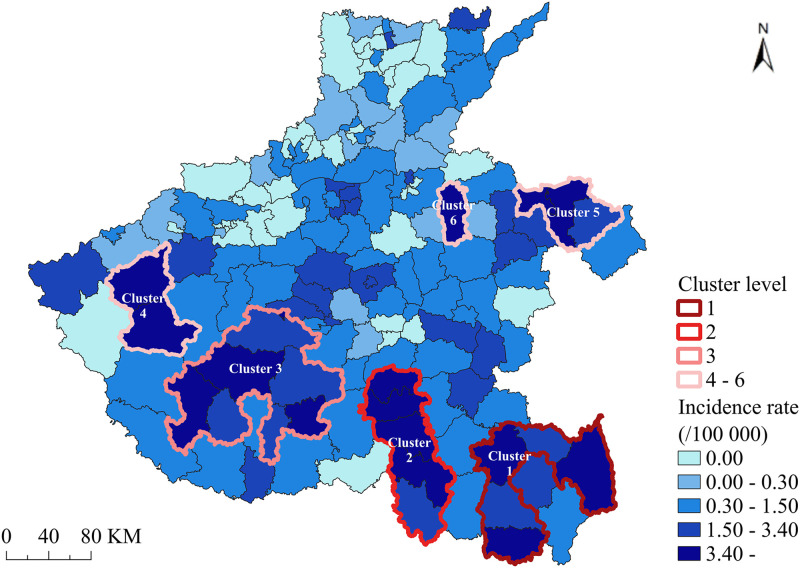
FleXScan-detected clusters on the measles incidence map for joint validation.

Using the FleXScan method, six statistically significant high-incidence cluster of measles in Henan Province in May 2009 was identified (p < 0.05), as shown in Fig 7. Table 2 presents the detected cluster, ranked by the LLR, with a higher LLR value indicating stronger clustering.

**Table 2:** Results of spatial scan statistics for measles clusters in Henan Province, May 2009.

Year	Cluster level	Number of sub-regions	Number of cases	Expected cases	Risk population	Log-likelihood ratio	Relative risk	p-value
2009	1	5	177	52.18	3489239	97.47	3.39	0.001
	2	5	146	41.21	2755655	84.12	3.54	0.001
	3	8	187	79.78	5334516	56.65	2.34	0.001
	4	2	53	11.45	765801	40.29	4.63	0.001
	5	3	102	42.36	2832577	31.35	2.40	0.001
	6	1	52	15.65	1046626	26.57	3.32	0.001

The clusters identified by the FleXScan method were overlaid onto the measles incidence choropleth map of Henan Province for joint validation, as shown in Fig 7. Overall, the high-incidence clusters detected by FleXScan are largely consistent with the areas of elevated incidence on the choropleth map, indicating that the method reliably captures spatial clustering patterns.

**Fig 8:**
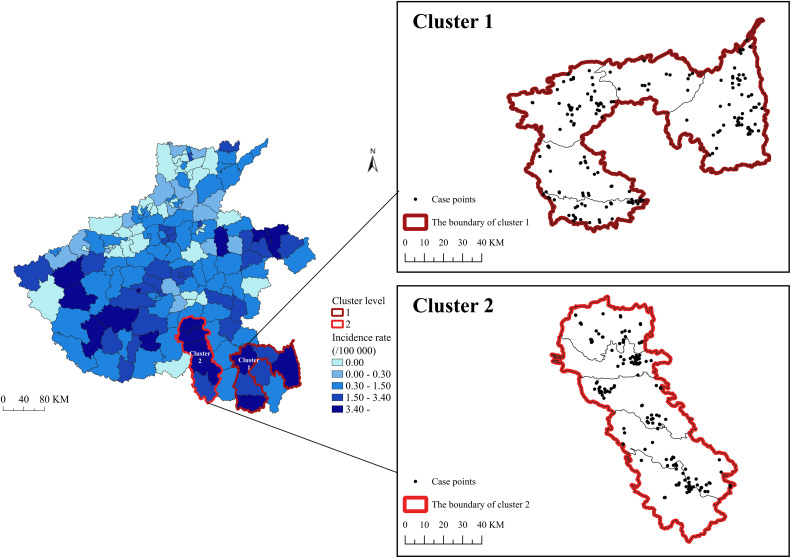
Dot cartograms of measles cases in two high-risk clusters.

Fig 8 presents the dot cartogram of case points within the two most likely clusters, revealing the internal spatial patterns of the clusters and highlighting potential high-risk cores. Due to the relatively small population differences among counties in Henan Province, the areas and shapes of Cluster 1 and Cluster 2 show only minor changes in the dot cartogram. Multiple distinct aggregations of case points are observed within both clusters. However, as the dataset covers only a single month and the number of cases is limited, accurately identifying high-risk cores within the clusters remains constrained.

## 5. Discussion and conclusions

This study developed an analytical framework integrating disease mapping with spatial scan statistics to identify and validate spatial clustering patterns of diseases. The framework enables multilevel analysis, from observing overall distribution and detecting significant clusters to identifying local high-risk cores. The proposed “exploration–validation–refinement” workflow highlights the complementary role of visualization and spatial analysis in investigating disease spatial patterns.

Disease spatial clustering is complex, and accurate analysis provides a theoretical basis for subsequent research on disease etiology and for developing effective control strategies, whereas inaccurate analysis may lead to ineffective interventions and even negative social and economic impacts [38]. Choropleth maps visually depict the overall spatial distribution of diseases, providing a basis for identifying potential high-risk areas, although they may be subject to visual bias due to differences in areal size and the MAUP. The FleXScan method effectively detects statistically significant clusters, generally consistent with the visualization patterns. Within the clusters, dot cartograms reveal internal heterogeneity, facilitating the localization of high-risk cores and supporting targeted interventions.

The framework was validated using both synthetic data and real measles case data, confirming the applicability of combining qualitative visualization with quantitative spatial analysis. In the synthetic data, dot cartograms demonstrated clear advantages in areas with large population density differences, compensating for limitations of conventional choropleth maps. For instance, in Area 04, although the incidence rate and areal size suggest a high visual weight in a choropleth map, the population was sparse and case counts were low; the dot cartogram more accurately represented this discrepancy. However, in the real measles data, the advantages of dot cartograms were less apparent, primarily due to relatively small population differences among counties in Henan Province. Furthermore, the data only cover one month, with a limited number of cases, and case points are not precise locations (randomly distributed within townships), which constrains the accurate identification of spatial clustering.

These findings indicate that the framework can effectively identify spatial clusters when supported by high-precision case data. However, the framework’s performance depends on data accuracy, and detailed case information is often difficult to obtain due to privacy restrictions. Additionally, this study only employed choropleth maps, point maps, and dot cartograms; no single mapping method can comprehensively represent all disease-related information and its uncertainties. Therefore, a comprehensive presentation of disease spatial patterns requires the integration of multiple mapping methods to provide complementary insights.

## Supporting information

S1 File Synthetic data. Example Shapefile containing the boundaries and attribute data of 10 artificially constructed synthetic regions, not corresponding to any real geographic units or disease events.
(ZIP)

## Abbreviations

GIS: Geographical Information Systems
MLCs: Most Likely Clusters
LLR: Log-likelihood Ratio

## References

[1] Paul E, Daniel W. Spatial epidemiology: current approaches and future challenges. Environ Health Perspect. 2004;112(9):998–1006. 15198920 10.1289/ehp.6735 PMC1247193

[2] Shrestha S, Bauer CXC, Hendricks B, Stopka TJ. Spatial epidemiology: an empirical framework for syndemics research. Soc Sci Med. 2022;295:113352. doi: 10.1016/j.socscimed.2020.113352 32950331 PMC7962030

[3] Tao MT. Using cartograms in disease mapping. Sheffield: The University of Sheffield; 2010.

[4] Harden SR, Schuurman N. Geospatial science and health: overview of data and methods. In: Understanding cancer prevention through geospatial science: putting cancer in its place; 2024. p. 67–93.

[5] Friendly M. A brief history of data visualization. In: Handbook of data visualization. Berlin, Heidelberg: Springer; 2008. p. 15–56.

[6] Merrill DW. Density equalizing map projections (cartograms) in public health applications. Berkeley: University of California; 1998.

[7] Murad A, Khashoggi BF. Using GIS for disease mapping and clustering in Jeddah, Saudi Arabia. ISPRS Int J Geo-Inf. 2020;9(5):328.

[8] Levison ME, Haddon W Jr. The area adjusted map. an epidemiologic device. Public Health Rep (1896). 1965;80(1):55–9. doi: 10.2307/4592353 14255452 PMC1919554

[9] Forster F. Use of a demographic base map for the presentation of areal data in epidemiology. Br J Prev Soc Med. 1966;20(4):165–71. doi: 10.1136/jech.20.4.165 5969679 PMC1059049

[10] Harris R, Charlton M, Brunsdon C. Mapping the changing residential geography of White British secondary school children in England using visually balanced cartograms and hexograms. J Maps. 2018;14(1):65–72. doi: 10.1080/17445647.2018.1478753

[11] Howe GM. Some recent developments in disease mapping. R Soc Health J. 1970;90(1):16–20. doi: 10.1177/146642407009000110 5504389

[12] Bithell JF. A classification of disease mapping methods. Stat Med. 2000;19(17–18):2203–15. doi: 10.1002/1097-0258(20000915/30)19:17/18<2203::aid-sim564>3.0.co;2-u 10960848

[13] Kronenfeld BJ, Wong DWS. Visualizing statistical significance of disease clusters using cartograms. Int J Health Geogr. 2017;16(1):19. doi: 10.1186/s12942-017-0093-9 28506288 PMC5433035

[14] Kronenfeld BJ, Yoo K. Effectiveness of animated choropleth and proportional symbol cartograms for epidemiological dashboards. Cartogr Geogr Inf Sci. 2023;51(2):330–46. doi: 10.1080/15230406.2023.2264755

[15] Kobakian S, Cook D, Roberts J. Mapping cancer: the potential of cartograms and alternative map displays. Ann Cancer Epidemiol. 2020;4:9–9. doi: 10.21037/ace-19-31

[16] Zhang C, Luo L, Xu W, Ledwith V. Use of local Moran’s I and GIS to identify pollution hotspots of Pb in urban soils of Galway, Ireland. Sci Total Environ. 2008;398(1–3):212–21. doi: 10.1016/j.scitotenv.2008.03.011 18440599

[17] Kulldorff M. A spatial scan statistic. Commun Stat - Theory Methods. 1997;26(6):1481–96. doi: 10.1080/03610929708831995

[18] Kulldorff M, Huang L, Pickle L, Duczmal L. An elliptic spatial scan statistic. Stat Med. 2006;25(22):3929–43. doi: 10.1002/sim.2490 16435334

[19] He R, Zhu B, Liu J, Zhang N, Zhang W-H, Mao Y. Women’s cancers in China: a spatio-temporal epidemiology analysis. BMC Womens Health. 2021;21(1):116. doi: 10.1186/s12905-021-01260-1 33743648 PMC7981806

[20] Li M, Liu Y, Yan T, Xue C, Zhu X, Yuan D, et al. Epidemiological characteristics of mumps from 2004 to 2020 in Jiangsu, China: a flexible spatial and spatiotemporal analysis. Epidemiol Infect. 2022;150:1–26. doi: 10.1017/S095026882200067X 35393005 PMC9074115

[21] Wang L, Li Y, Chen J, Zhang D, Zhang Z, Li X. Spatiotemporal distribution and detection of spatial clusters of tuberculosis in Hubei Province, China using FleXScan (2017-2023). BMC Public Health. 2025;25(1):2758. doi: 10.1186/s12889-025-23620-4 40813658 PMC12351876

[22] Wang L, Li X, Zhang Z, Yuan H, Lu P, Li Y. Comparing circular and flexibly-shaped scan statistics for disease clustering detection. Front Public Health. 2025;12:1432645. doi: 10.3389/fpubh.2024.1432645 39845679 PMC11750662

[23] Mas J-F, Pérez-Vega A. Spatiotemporal patterns of the COVID-19 epidemic in Mexico at the municipality level. PeerJ. 2021;9:e12685. doi: 10.7717/peerj.12685 35036159 PMC8711283

[24] Tango T. Spatial scan statistics can be dangerous. Stat Methods Med Res. 2021;30(1):75–86. 33595399 10.1177/0962280220930562

[25] Tango T, Takahashi K. A flexibly shaped spatial scan statistic for detecting clusters. Int J Health Geogr. 2005;4:11. doi: 10.1186/1476-072X-4-11 15904524 PMC1173134

[26] Tango T, Takahashi K. A flexible spatial scan statistic with a restricted likelihood ratio for detecting disease clusters. Stat Med. 2012;31(30):4207–18. doi: 10.1002/sim.5478 22807146

[27] Soetens L, Hahné S, Wallinga J. Dot map cartograms for detection of infectious disease outbreaks: an application to Q fever, the Netherlands and pertussis, Germany. Eurosurveillance. 2017;22(26):30562. doi: 10.2807/1560-7917.ES.2017.22.26.30562 28681721 PMC5779165

[28] Plaza-Rodríguez C, Appel B, Kaesbohrer A, Filter M. Discussing state-of-the-art spatial visualization techniques applicable for the epidemiological surveillance data on the example of Campylobacter spp. in raw chicken meat. Zoonoses Public Health. 2016;63(5):358–69. doi: 10.1111/zph.12231 26404056

[29] Gastner MT, Newman MEJ. From The Cover: Diffusion-based method for producing density-equalizing maps. Proc Natl Acad Sci U S A. 2004;101(20):7499–504. doi: 10.1073/pnas.0400280101 15136719 PMC419634

[30] Gastner MT, Seguy V, More P. Fast flow-based algorithm for creating density-equalizing map projections. Proc Natl Acad Sci U S A. 2018;115(10):E2156–64. doi: 10.1073/pnas.1712674115 29463721 PMC5877977

[31] Selvin S, Merrill D, Schulman J, Sacks S, Bedell L, Wong L. Transformations of maps to investigate clusters of disease. Soc Sci Med. 1988;26(2):215–21. doi: 10.1016/0277-9536(88)90242-0 3258072

[32] Schulman J. The statistical analysis of density equalized map projections. Berkeley: University of California; 1986.

[33] Schulman J, Selvin S, Merrill DW. Density equalized map projections: a method for analysing clustering around a fixed point. Stat Med. 1988;7(4):491–505. doi: 10.1002/sim.4780070406 3368676

[34] Merrill DW. Use of a density equalizing map projection in analysing childhood cancer in four California counties. Stat Med. 2001;20(9–10):1499–513. doi: 10.1002/sim.686 11343370

[35] Khalakdina A, Selvin S, Merrill DW, Erdmann CA, Colford JM Jr. Analysis of the spatial distribution of cryptosporidiosis in AIDS patients in San Francisco using density equalizing map projections (DEMP). Int J Hyg Environ Health. 2003;206(6):553–61. doi: 10.1078/1438-4639-00245 14626902

[36] Dean AG. Population-based spot maps: an epidemiologic technique. Am J Public Health. 1976;66(10):988–9. doi: 10.2105/ajph.66.10.988 970517 PMC1653441

[37] Ma Y, Yin F, Zhang T, Zhou XA, Li X. Selection of the maximum spatial cluster size of the spatial scan statistic by using the maximum clustering set-proportion statistic. PLoS One. 2016;11(1):e0147918. doi: 10.1371/journal.pone.0147918 26820646 PMC4731069

[38] Xie Y, Shekhar S, Li Y. Statistically-robust clustering techniques for mapping spatial hotspots: a survey. ACM Comput Surv. 2022;55(2):1–38. doi: 10.1145/3487893

